# No molecular detection of tick-borne pathogens in the blood of patients with erythema migrans in Belgium

**DOI:** 10.1186/s13071-021-05139-w

**Published:** 2022-01-20

**Authors:** Laurence Geebelen, Tinne Lernout, Katrien Tersago, Sanne Terryn, Joppe W. Hovius, Arieke Docters van Leeuwen, Steven Van Gucht, Niko Speybroeck, Hein Sprong

**Affiliations:** 1 Scientific Directorate of Epidemiology and Public Health, Sciensano, Brussels, Belgium; 2 Institute of Health and Society (IRSS), Université Catholique de Louvain, Woluwe-Saint-Lambert, Belgium; 3 Flemish Agency for Care and Health, Brussels, Belgium; 4 Scientific Directorate of Infectious Diseases in Humans, Sciensano, Brussels, Belgium; 5 Amsterdam UMC Multidisciplinary Lyme Borreliosis Center, Amsterdam University Medical Centers (UMC), Location AMC, Amsterdam, The Netherlands; 6 Centre for Infectious Disease Control, National Institute for Public Health and Environment (RIVM), Bilthoven, The Netherlands

**Keywords:** Tick-borne pathogens, *Neoerhlichia mikurensis*, Lyme borreliosis, Erythema migrans, *Ixodes ricinus*, *Neoehrlichosis*

## Abstract

**Background:**

A number of tick-borne pathogens circulate in the Belgian tick population in addition to the causative agent of Lyme borreliosis. However, so far, only a few patients with tick-borne diseases other than Lyme borreliosis have been reported in Belgium. The aim of this study was to investigate the occurrence of other human tick-borne infections in Belgium and their possible clinical manifestation.

**Methods:**

Patients with fever (> 37.5 °C) after a tick bite or those with erythema migrans (EM) were included in the study. EDTA-blood samples were screened for the presence of DNA from *Borrelia burgdorferi* sensu lato, *Borrelia miyamotoi*,* Anaplasma phagocytophilum*, *Neoehrlichia mikurensis*, spotted fever group rickettsiae (genus* Rickettsia*), *Babesia* spp., *Bartonella* spp., *Spiroplasma ixodetis* and tick-borne encephalitis virus, using multiplex PCR methods. A questionnaire on, among others, demographics and clinical symptoms, was also filled in.

**Results:**

Over a period of 3 years, 119 patients with EM and 14 patients with fever after a recent tick bite were enrolled in the study. Three samples initially tested positive for *N. mikurensis* by quantitative PCR (qPCR), but the results could not be confirmed by other PCR methods, and repetition of the DNA extraction procedure and qPCR test was not successful. The qPCR test results for the other tick-borne pathogens were negative.

**Conclusions:**

In general, only a few patients with fever after a tick bite could be identified. Although no tick-borne pathogens were detected, their occurrence cannot be excluded based on the limited number of patients and the limitations inherent to current methodologies. This study underscores the possibility of false-positive PCR results and the necessity for the development of multiple independent tools for the sensitive and specific detection of emerging tick-borne pathogens.

**Graphical Abstract:**

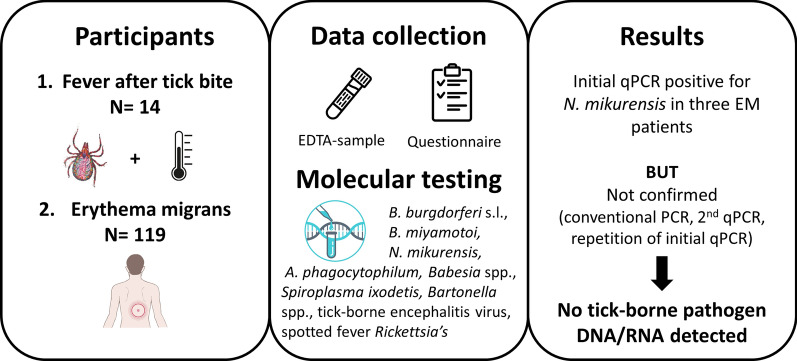

**Supplementary Information:**

The online version contains supplementary material available at 10.1186/s13071-021-05139-w.

## Background

Lyme borreliosis (Lyme disease), caused by spirochetes from the *Borrelia burgdorferi* sensu lato (s.l.) complex, is the most common tick-borne disease in Europe and North America [1]. The tick *Ixodes ricinus* is the main vector of Lyme borreliosis in Europe, and is known to transmit several other infectious diseases as well, including tick-borne encephalitis (TBE), anaplasmosis and babesiosis [2, 3]. Some less known pathogens transmitted by these ticks, such as *Borrelia miyamotoi* and *Neoehrlichia mikurensis*, have more recently been found to be pathogenic to humans although only a limited number of cases have been described in Europe [4–6]. For other less known pathogens, such as *Rickettsia helvetica*, the pathogenicity remains unresolved [7–9]. A Belgian study in 2017 [10] detected several potential pathogens in *I. ricinus* ticks removed from humans, with a reported prevalence of 14% for *B. burgdorferi* s.l.; 1.5% for *Babesia* spp., 1.8% for *Anaplasma phagocytophilum*, 2.4% for *B. miyamotoi*, 2.8% for *N. mikurensis*, 6.8% for *R. helvetica*, and also 13.5% for *Spiroplasma ixodetis* (Additional file 1: Database S1). The same study identified *Rickettsia raoultii* in two out of five *Dermacentor reticulatus* ticks, and co-infections were reported in 3.9% of the screened ticks [10]. In humans, many of these infections may occur asymptomatically or may cause non-characteristic, self-limiting, flu-like symptoms for which patients may not seek medical assistance. However, some may occasionally cause severe disease, especially in immunocompromised patients [3]. In Belgium, no or only few cases of human tick-borne infections other than Lyme borreliosis have been reported [10]. Although TBEV was not detected in the ticks screened in that 2017 study, the first two human cases, classified as possible and probable autochthonous, respectively, were diagnosed in 2018 [10]. For human granulocytic anaplasmosis, only few confirmed cases have been diagnosed, but every year there are between 10 and 20 probable cases based on symptoms and serology, and underdiagnosis is suspected due to difficulties in diagnosis and lack of awareness [11, 12]. Underdiagnosis also probably accounts for the lack of reports on several other tick-borne pathogens. Take, for example, human babesiosis: a Belgian study showed the presence of antibodies against three *Babesia* species in blood collected from persons presenting with symptoms after a tick bite, yet no human clinical cases have been reported [13]. In addition, although the relapsing fever spirochete *B. miyamotoi* is present in ticks, no cases of *B. miyamotoi* disease, or of neoehrlichiosis, caused by infections with the intracellular bacterium *N. mikurensis*, have been reported in Belgium [14, 15]. The latter, previously called ‘*Candidatus* N. mikurensis’, was only recently cultured successfully which is why it lost its candidatus status [14]. For the tick-borne spotted fever rickettsiae, such as *R. helvetica*, *R. monacensis* or *R. raoultii*, no confirmed autochthonous infections or cases have been reported in Belgium [10]. Although *Bartonella* spp. DNA can be found in *I. ricinus*, it remains uncertain whether they are a relevant vector for the transmission of the disease [16, 17]. Bartonellosis, mainly cat-scratch disease caused *by Bartonella henselae*, has been regularly diagnosed in Belgium [18]. Similarly, although found in ticks, the transmission of *Spiroplasma ixodetis*, causing intraocular infection in newborns, by ticks is still unconfirmed [19]. Only a handful of human infections have been described, but so far never in Belgium [20, 21].

In general, the incidence and severity of tick-borne infections other than Lyme borreliosis and TBE are largely unknown and the public health implications remain unclear. Fever has been described as one of the most common symptoms associated with several of these other tick-borne infections [3, 9]. Nevertheless, this finding is often based on case reports only [9]. Furthermore, concurrent infection with *Anaplasma* spp. or *Babesia* spp. might exacerbate the course of Lyme borreliosis, but information on the impact of other co-infections is lacking [22–24]. Due to the mild non-specific symptomatology of many tick-borne diseases, as well as a low awareness among physicians and patients and a lack of routinely available diagnostic tests, it is possible that several of these diseases are underdiagnosed [25]. In addition, many patients cannot remember a tick bite, complicating diagnosis.

The aim of this study was to evaluate the presence of tick-borne pathogens other than *Borrelia* spirochetes using PCR techniques in blood collected from patients with a recent tick bite and fever, a common symptom of these other infections, and in patients with an erythema migrans (EM), the most common clinical manifestation of Lyme borreliosis. The study also aimed to investigate the ability of these pathogens to cause clinical disease, presenting as fever or other symptoms to general practitioners (GPs).

## Methods

### Study design and participant enrollment

Between June 2016 and August 2019, patients with fever (> 37.5 °C, reported by the patient or GP) within 1 month after a tick bite or with an EM (including multiple EM) were included in the study by a network of GPs set up in areas endemic for tick bites and Lyme borreliosis in Belgium. GPs were invited to participate in the study by emails sent through the GP associations from these areas and by personal invitation by post. At the beginning of the study the network consisted of about 50 GPs; this number expanded during the study period to about 200 GPs from 2018 onwards. After registration, participating GPs received packages by mail providing information on the study, including guidelines for the diagnosis of an EM [26], blood sampling material and the inclusion questionnaire for both groups of patients. Patients were included prospectively around the time of their diagnosis in order to collect an EDTA-blood sample of 6 ml during the acute phase of illness and before antibiotic treatment was initiated. Patients aged < 18 years and pregnant women were not eligible for inclusion in the study. Potential participants were also excluded when the geographical location where the tick bite occurred (if known) was not in Belgium, and when the EM diameter was < 5cm and the patient did not recall a tick bite or the delay in appearance of EM was <2 days (if date of tick bite was known) [26]. The questionnaire consisted of a first part to be filled out together by the patient and GP, which contained questions on the diagnosis, treatment, comorbidities (including immunocompromising illness or treatments), antibiotic use in the month before the blood sample was taken and symptoms at diagnosis (group with fever after tick bite), and a second part to be completed by the patients themselves on demographics, symptoms at diagnosis (EM patients) and exposure to tick bites.

### Sample preparation and molecular testing

The whole blood-EDTA samples were sent by the GPs to the Belgian Health Institute Sciensano, where they were aliquoted and stored at − 80 °C. Aliquots were later sent in batches to the Laboratory for Zoonoses and Environmental Microbiology, National Institute for Public Health and Environment (RIVM), in the Netherlands. Total nucleic acid extraction from the EDTA-blood samples was performed using a robotic workstation (MagNA Pure Compact Extraction Robot; Roche, Basel, Switzerland) on 200 μl of EDTA-plasma (Nucleic Acid Isolation Kit I; Roche) in a diagnostic laboratory setting, following the manufacturer’s instructions. To detect potential cross-contamination, negative controls were included in each batch of extractions. All samples were analyzed with different (multiplex) real-time PCR assays, with each assay based on various genes specific for the microorganism of interest, including *B. burgdorferi* s.l. (two gene targets: *ospA* and *flab*) [27], *B. miyamotoi* (target: *flagellin*) [28], *A. phagocytophilum* (target: *msp2*) [29, 30], *N. mikurensis* (target: *groEL*) [31], spotted fever group rickettsiae (members of genus* Rickettsia*; target: *gltA*) [32], *R. helvetica* (target: *gltA*) [33] *Bartonella* spp. (target: *ssrA*) [34], *Babesia microti* (target:* 18S rRNA*) [35] and *Babesia* species from the* Babesia* senso stricto clade (target:* 18S rRNA*) [36, 37]. A quantitative PCR (qPCR) assay for the detection of *S. ixodetis* was newly developed using primers targeting a 170-bp fragment of the RNA polymerase subunit, Spir_rpoB-F (5′-TGT-TGG-ACC-AAA-CGA-AGT-TG-3′) and Spir_rpoB-F (5′-CCA-ACA-ATT-GGT-GTT-TGG-GG-3′), and probe 5′-(Atto425)-GCT-AAC-CGT-GCT-TTA-ATG-GG(BHQ1)-3′ [38]. Ticks removed from humans in all Belgian provinces, which had been collected and analyzed in a previous study, were also tested for *S. ixodetis* [10]. Of 1515 ticks tested, 204 were positive (results shown in Additional file 1: Database S1). These qPCRs were carried out on a LightCycler 480 System (Roche Diagnostics Nederland B.V, Almere, the Netherlands) in a final reaction volume of 20 μl containing iQ multiplex Powermix, 3 μl of sample, 0.2 μM of all primers and different concentrations for the different probes. Positive plasmid controls and negative water controls were used on every plate tested. The nucleic acid extractions of the EDTA-blood samples were also tested for the presence of tick-borne encephalitis virus (TBEV) RNA. For the latter PCR analysis, a multiplex reverse transcription real-time PCR was performed as described by Lindblom et al. [39]. In short, reactions were carried out in a final reaction volume of 20 μl that contained TaqMan Fast Virus 1-Step Master Mix (Thermo Fisher Scientific, Waltham, MA, USA) to which 5 μl of sample, 0.2 μM of all primers and 0.2 μM probes were added. An internal control was added to all samples. The amplification was performed on a Roche LightCycler 480 System set at the following cycling program: a 20-min reverse transcription step at 50 °C; followed by denaturation at 95 °C for 30 s; and then 50 cycles of 95 °C/10 s and 60 °C/30 s. Conventional PCRs were performed on all samples that were found to be positive by the real-time PCR as confirmation for one or more targets, followed by Tris-borate-EDTA-agarose gel electrophoresis as previously described [40]. In addition, samples positive in the real-time PCR for *N. mikurensis* were sent by normal mail to the Department of Natural Science and Environmental Health at the University of South-Eastern Norway to be analyzed with a second real-time PCR targeting another fragment of the *groEL* gene, as described previously [41]. More specifically, the SYBR-green PCR set-up was used, and either 5 μl or 0.5 μl of DNA lysate was added in a final reaction volume of 25 μl. EDTA-blood samples originally testing positive for *N. mikurensis* were extracted once again at the RIVM in the Netherlands with the Qiagen DNeasy Blood and Tissue Kit according to the manufacturer’s protocol (Qiagen, Hilden, Germany) and tested again with the qPCR assay for *N. mikurensis* (target: *groEL*) [31]. To minimize contamination and false-positive samples, the DNA/RNA extraction, PCR mix reparation, sample addition, and (q)PCR analyses were performed in separated air-locked, dedicated laboratories.

## Results

### Patient characteristics and symptoms

**Fig. 1:**
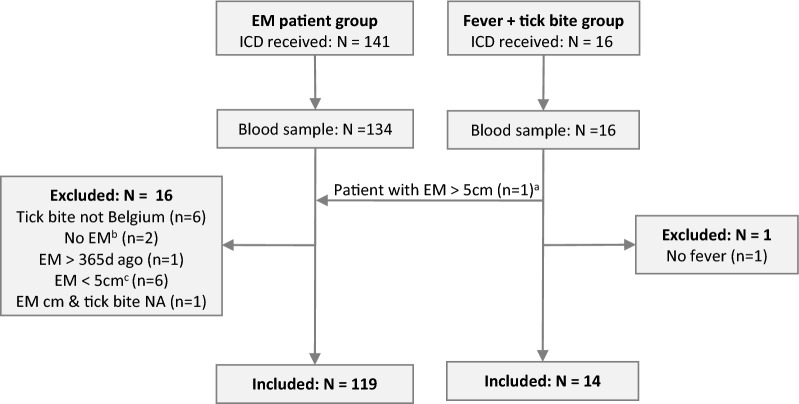
Flow chart of patient inclusion. ^a^One patient was included in the group of patients with fever after a recent tick bite but was switched to the EM group as the GP also reported multiple EM. ^b^EM diagnosis was later disputed by the patient’s GP. ^c^EM < 5 cm without recall of tick bite (*n* =  5) or a known delay in appearance of < 2 days (*n* = 1). Abbreviations: d, days; EM, erythema migrans; GP, general practitioner; ICD, informed consent document; NA, missing data

In total, blood samples were collected from 150 patients recruited by the GP network, of whom 17 patients were excluded from the study (Fig. 1). Of the 133 patients who were included in the study, 119 had an EM (89.5%) and only 14 patients had fever after a recent tick bite without an EM.

Among these 133 patients, 62% and 43% of the EM patient group and fever after tick bite group, respectively, were female. The mean age of all patients was 53 (range 18–95) years. The majority of patients (74%) included in the study had reported to their GP between June and August. Of the EM patients for whom data were available (*n* = 118), 64% could remember a tick bite that had occurred at a median of 14 days before the diagnosis (range 1–102 days). Time since first notice of the EM ranged from 0 to 90 (median 7.5) days (data missing for 11 patients). For the patients with fever after a tick bite (for whom remembering the bite was an inclusion criterion), the bites occurred a median of 20 (range 8–60) days before the visit to the GP. In 11 EM patients (9%), the EM diameter was < 5 cm; for those with EM of a larger diameter, the median diameter was 10 (range 5–30) cm (data missing for 5 patients). In total, 10 GPs reported that their patient had taken an antibiotic in the month before the blood sample was drawn (data missing for 2 patients); of these, in six patients the antibiotic was considered to have the potential to impact the PCR result, and in the other four patients the antibiotic was taken > 10 days before the tick bite occurred (*n* = 3) or concerned an antibiotic expected to be ineffective (*n * =  1). Symptoms reported at inclusion in the study are described in Table 1.

**Table 1:** Symptoms reported at inclusion by the two groups of patients: those with fever after a tick bite and those with an erythema migrans

Symptom^a^	Fever + tick bite group (*N* = 14)	EM patient group (*N* = 119)
	*n*/*N*^b^ (%)	*n*/*N*^b^ (%)
Fever at inclusion (> 37.5 °C)	13/13 (100%)	5/118 (4%)
Fever, measured	8/13	3/118
Fever, not measured	5/13	2/118
Unknown	0/13	15/118
Fatigue	14/14 (100%)	41/115 (36%)
Night-sweats	10/13 (77%)	18/115 (16%)
Muscle pain (local/general)	10/14 (71%)	33/110 (30%)
Headache	10/14 (71%)	29/116 (25%)
Joint pain	8/14 (57%)	29/113 (26%)
Neck pain	7/13 (54%)	24/114 (21%)
Swollen joints	3/14 (21%)	10/113 (9%)
Cognitive difficulties	3/14 (21%)	17/115 (15%)
Nausea	2/14 (14%)	10/116 (9%)

*EM* Erythema migrans

^a^For the group of patients with fever after a recent tick bite, symptoms are those since the tick bite; for the group of patients with an EM, symptoms are those in the 2 weeks before diagnosis of the EM or at diagnosis of the EM

^b^Total no. of patients with symptom/total no. of patients for whom information on this symptom is available

### Molecular detection of tick-borne pathogens

A total of three of the 133 (2.3%) blood samples tested positive in the multiplex qPCR for the presence of *N. mikurensis* DNA; the sigmoid curves for these three results were of normal shape and height but the cycle threshold (Ct) values were relatively high (37.2, 37.9 and 38.8, respectively), implying either low bacteremia or non-specific or cross-reactive fluorescence signals. Despite several attempts, the presence of *N. mikurensis* DNA could not be confirmed in the three qPCR-positive samples by conventional PCR, which is less sensitive than the multiplex qPCR (not shown). Therefore, DNA was re-extracted from the *Neoehrlichia*-positive blood samples and sent to a laboratory in Norway for confirmation—where the samples tested negative. When the initial multiplex qPCR was subsequently repeated, it was also negative. The DNA or RNA of other pathogens was not detected. The three samples initially positive for *N. mikurensis* were from two male and one female EM patients, of whom one was a young adult and two were aged > 80 years. The characteristics of these three patients are shown in Table 2. None reported suffering from an immunocompromising disease or using immunosuppressive drugs, and none reported fever, although this latter information was unavailable for one patient.

**Table 2:** Characteristics of the three erythema migrans patients who tested positive for *Neoehrlichia mikurensis* in the initial quantitative PCR test

Patient	Age (years)	Sex	Time between tick bite and T0	Symptoms at T0
#1	90+	Female	No recall of tick bite (30 days since start EM)	EM (diameter: 30 cm)
#2	80–84	Male	28 days	EM (diameter: 12 cm)
#3	20–24	Male	35 days	EM (diameter: 8 cm), general muscle pain, neck pain, fatigue, headache, nausea, cognitive difficulties (fever unknown)

*T0* Time of diagnosis by general practitioner, *EM* Erythema migrans

Patient #2 suffered from dementia. The information on subjective symptoms might have been incomplete

In nine of the 14 patients presenting with fever after a recent tick bite, the GP suspected Lyme borreliosis as the underlying cause, three reported an (atypical) erythema of < 5 cm. No pathogens were detected in these blood samples.

## Discussion

Prospective studies investigating the occurrence of tick-borne infections other than Lyme borreliosis in persons bitten by ticks and their ability to cause clinical disease remain rare [9, 40, 42–44]. By including patients with fever after a recent tick bite in the present study, our aim was to search for these pathogens in a group of patients expected to be at a higher risk for infection. However, despite a broad molecular screening for various pathogens in this study, no tick-borne infections could be detected in the 14 patients included in this group. Possible explanations are that the fever was caused by another infectious agent unrelated to the tick bite or by another tick-borne pathogen not searched for, that the detection methods for the current pathogens were insufficient (see below) or that an antibiotic treatment was taken before sample collection. All 14 patients in this group (fever after a recent tick bite) experienced other symptoms in addition to fever, of which fatigue, night sweats, muscle pain and headache were the most common.

In the group of patients with an EM, we also did not detect any tick-borne co-infections. These results are not completely unexpected, given the limited number of patients in the study and the inherent limitations of the detection methods used. Prospective studies in other countries that have also analyzed similar tick-borne pathogens with PCR also found only few infections: a study in the Netherlands on 626 persons with a tick bite or an EM and a study in Austria on 489 tick-bitten persons both reported that about 2.6% of the participating patients were positive for any of the pathogens researched [40, 42]. However, a Norwegian study involving 70 patients with symptoms, mainly EM, found bacterial DNA in 14% of the patients’ samples [44]. Even though no tick-borne pathogens were detected in the blood samples in our study, their presence cannot be excluded*.* The PCR tests may not be sensitive enough to detect cases; as such, additional serological tests could be of value, as was the case in a recent study in the Nordic countries, which found more infections based on seroconversion than by PCR tests [43]. In addition, tissue tropism of the pathogens and timing of the collection of the blood sample have to be taken into account for all tick-borne diseases when PCR is used as a diagnostic method [11, 45]. *Noehrlichia mikurensis*, *Babesia* spp., *B. miyamotoi* and *A. phagocytophilum* are expected to be found in the blood while *B. burgdorferi* s.l. on the other hand is not, which explains why none of the EM patients in the study tested positive for the latter [6, 11, 14, 46]. Taken together, we were therefore unable to assess whether the fever in patients with a recent tick bite could have been caused by infection with *B. burgdorferi* s.l. For some pathogens PCR assays are most sensitive during the acute phase of illness, and the detection period may be short. A Russian study found that *B. miyamotoi* can only be detected by PCR during the first 3 days of acute disease [47], and *A. phagocytophilum* is expected to be detectible for < 2 weeks after disease onset [11]. Although our expectation was that patients with fever after a recent tick bite would be in this acute phase of infection, for eight of the 13 patients for whom such data were available (missing data on 1 patient), symptoms first appeared > 3 days before the consultation with the GP, and for five patients they first appeared ≥ 2 weeks before inclusion. Patients with an EM could have not yet reached or already passed this acute phase since for those remembering their tick bite, it took place at a median of 14 days before inclusion (range 1–102 days). Finally, antibiotic treatment before sample collection could result in a negative PCR result; since this is not necessarily the case, we still included these patients in the study. For six patients it was reported that they had taken at least one dose of an antibiotic before sample collection (data missing on 2 patients), potentially impacting the PCR result. One of the patients who had initially tested positive had taken an antibiotic in the week before the blood draw, but no effect was expected.

In addition to the possibility of false negativity of PCR results, the current study also emphasizes the possiblity of false positivity of PCR results. Three samples were positive for *N. mikurensis* in the first qPCR performed, yet all three had high Ct values, indicating low levels of *N. mikurensis* DNA in the sample. Although there were no irregularities or signs of contamination in the first qPCR result (bands were of the correct size, no secondary bands, positive and negative controls correctly analyzed) and previous testing with this qPCR method suggested that it is specific [31], the results could not be confirmed by a conventional PCR. As qPCR methods are generally more sensitive than conventional PCR methods and, in our experience, samples with high Ct values generally remain negative by conventional PCR, this result was not completely unexpected. However, this result may also represent non-specific or cross-reactive fluorescence signals, and therefore the samples were further tested with the SYBR-green qPCR method from Norway, which also did not confirm the positive test results. This second qPCR protocol uses different primer pairs and yields a longer PCR product than the first qPCR. The discrepancies between the first and second qPCR might therefore have been due to differences in sensitivity and specificity, caused, for example, by the reaction conditions or by some critical point mutations that have been described to occur in the *groEL* gene [41]. To verify this further, the first multiplex qPCR was repeated, and this time all three samples were negative. Although some DNA degradation might have occurred during storage or transport of the DNA samples, as the additional testing was performed 2 years after the initial testing, the results were ultimately regarded as being negative. The discrepancies in the PCR test results in this study are unsettling and underscore the need for complementary diagnostic tests, such as serological tools, for *N. mikurensis* in the future. The current lack of these tests complicates research on the presence and possible pathogenicity of these bacteria. The recent cultivation of *N. mikurensis* can accelerate the development of these tests [14]. The possibility of false positive results has previously also been emphasized for *B. miyamotoi* [48], highlighting the need for additional independent confirmation of non-routine PCR results for tick-borne pathogens in general.

The detection of *N. mikurensis* DNA by PCR has been reported in several studies. Similar to our results, a study in symptomatic patients in Poland found that three patients were positive for *N. mikurensis* by PCR (conventional) testing, but ultimately no infection was reported as the results could not be confirmed with sequencing [49]. However, in other studies, such as the prospective studies from the Netherlands, Austria and Norway mentioned above, *N. mikurensis* was detected in patients, and identified as the most commonly found pathogen, with a prevalence of 1.1, 2.3 and 10% in these three studies, respectively [40, 42, 44]. The positive patients were asymptomatic or had non-characteristic symptoms, such as fever, headache, arthralgia, myalgia and malaise. In addition, *N. mikurensis* has been detected in two of 102 persons bitten by ticks in a study in Sweden [43, 50], in five asymptomatic foresters in Poland [51] and in 12 subclinical/asymptomatic immunosuppressed patients in Norway [52]. Clinical cases have been reported in Europe, originating from Germany, Czech Republic, Sweden and Switzerland [4, 14, 53, 54].

Only 14 patients with fever after a recent tick bite could be included in this study over a period of > 3 years, whereas the number of patients with an EM was almost 10-fold higher. In addition, only five of the 119 EM patients reported fever (unknown in 15 EM patients). There are a number of different explanations that possibly explain this reported low prevalence of fever after a tick bite. First, it is noted that the most common tick-borne disease is Lyme borreliosis, for which fever (alone or with an EM) is rare [1, 55]. Also, the findings may suggest that other tick-borne diseases, in which fever are more common, do not often occur in Belgium. Finally, patients with fever probably do not relate this to a recent tick bite, and if the fever remains mild and is short-lived, they do not consult a physician. However, even though these cases do not impact the public health system and the disease burden of such cases is expected to be small more research is needed on whether they develop long-term sequelae and whether they would need treatment. Furthermore, when comparing the number of patients included in both groups, it has to be acknowledged that patients not remembering a tick bite could be included in the EM group (i.e. 36%), but not in the group of fever after a recent tick bite. In addition, it might have been more difficult for GPs to remember to ask a patient about a recent tick bite when fever was present than to include an EM patient in the study. However, to increase GPs’ awareness, each year, reminder emails were sent at the beginning of and halfway through the tick season, emphasizing the need to enroll both patient groups. There was no difference in the GP’s work load in terms of including patients of both groups in the study, but willingness to participate could have been higher for those patients with fever after a recent tick bite as the questionnaire was shorter and there was no follow-up, in contrast to the case of EM patients in part of another study [56].

Given the low number of patients with fever after a recent tick bite found in this study, it could be useful for future studies on infections other than Lyme borreliosis to extend inclusion criteria beyond fever, to include patients with other flu-like symptoms after a recent tick bite, in order to increase the sample size. It might be useful to include patients who were bitten by a tick > 1 month previously, in case *N. mikurensis* detection is aimed for, as it has been suggested in the literature that this bacterium persists in the blood for a longer period, even up to months [42, 50–52]. It is expected that, in the future, more tick-borne pathogens will be discovered due to the increase in molecular methods and availability of next-generation sequencing, of which the pathogenicity will have to be investigated [45].

## Conclusions

In this study, no tick-borne pathogen DNA/RNA was detected as an infection in blood samples of patients with an EM or fever after a recent tick bite in Belgium and no evidence of clinical disease caused by a tick-borne infection other than Lyme borreliosis was found. Although our results suggest that the occurrence of fever after a tick bite is low, at this point in time it remains impossible to determine the incidence, severity and public health risk of other tick-borne diseases in Belgium. In order to do so, knowledge on pathogenicity should first be increased, case definitions should be established and accurate diagnostic methodolgy, including serology, should be implemented. This study underscores the limitations and the possibilities of false positives by qPCR testing and the necessity for the development of multiple independent tools for the sensitive and specific detection of emerging tick-borne pathogens.

## Supplementary Information

**Additional file 1: Database S1.** Dataset of qPCR results Spiroplasma ixodetis.

## Data Availability

The dataset analyzed during the current study is available from the corresponding author on reasonable request.

## Abbreviations

Ct: Cycle threshold
EM: Erythema migrans
GP: General practitioner
s.l.: Sensu lato
TBE: Tick-borne encephalitis
TBEV: Tick-borne encephalitis virus
